# Effects of Different Environment-Friendly Gibberellic Acid Microcapsules on Herbicide Injury of Wheat

**DOI:** 10.3389/fpls.2022.915506

**Published:** 2022-07-11

**Authors:** Guisen Zhang, Tao Ma, Yong Cheng, Jianing Wang, Lang Liu, Baojun Zhang

**Affiliations:** ^1^College of Plant Protection, Shanxi Agricultural University, Jinzhong, China; ^2^Shanxi Inspection and Testing Center (Shanxi Institute of Standards and Metrology), Taiyuan, China; ^3^Bayer Crop Science (China) Co., Ltd., Beijing, China

**Keywords:** microcapsules, gibberellic acid, brassinolide, phytotoxicity, soluble powder

## Abstract

Environmentally friendly microcapsules are becoming more and more widely used due to the increasing demand for environmental safety in pesticides. To compare the impact of differences in wheat herbicide phytotoxicity of different gibberellic acid microcapsule suspensions, two microcapsule suspensions were separately formulated using the phase transfer method and the *in situ* polymerization method, and the key performance indicators are the size of microcapsules and the degree of encapsulation. Meanwhile, through field trials, the pharmacological and detoxification effects of different types of microcapsule suspensions on the herbicide methyldisulfuron in wheat fields were compared. The microcapsule suspension was prepared by the phase transfer method and the particle sizes D_10_, D_50_, and D_90_ are 0.990, 2.136, and 5.201 μm, respectively; the microcapsule suspension was prepared by the *in situ* polymerization, the particle sizes D_10_, D_50_, and D_90_ are 4.365, 8.547, and 16.782 μm, respectively. The encapsulation rate of the microcapsules prepared by the phase transfer method and the *in situ* polymerization method was 86.9% and 91.2%, being higher than 80%, the national standard for capsules. Meanwhile, the release rate conforms to first-order release kinetics in 0–4 days and zero-order release kinetics in 5–28 days. The plot trials' result showed that the detoxification effect of the microcapsules prepared by the *in situ* polymerization method was significantly better than the detoxification effect of the microcapsules prepared by the phase transfer method and the control agent. The growth index of wheat was higher than that of the untreated check after using the agent.

## Introduction

Microcapsule technology is widely used in global agricultural production because microcapsules are more environmentally friendly compared with other pesticide formulations, such as pesticide emulsion, wettable powder, and microemulsion. At present, inorganic or organic polymer materials are mainly coated on the surface of the active ingredient solution *via* physical and chemical methods to form solid particles with a certain shape. The diameter of such solid particles is generally between 10 and 1,000 microns (Cui et al., 2018), and the release of active ingredients occurs mainly through two mechanisms: osmotic diffusion and capsular rupture (Wang, 2009). Therefore, microcapsule technology can provide controlled release, prolong residual efficacy, and improve products' storage stability, and all the features can provide a significant value to pesticides applied in agricultural production, which has a high application value (Liu et al., 2018).

Gibberellic acid is a plant growth regulating hormone with good performance and widely used in global agricultural production. Gibberellic acid can provide excellent performance in promoting plant growth and cell differentiation, delaying senescence, and increasing crop tolerance. Gibberellic acid applied during the wheat growth stage promotes early wheat maturity, improves wheat quality and yield, and at the same time can improve wheat crop tolerance (Liu et al., 2015; Chen, 2018; Yu et al., 2019). However, the performance of gibberellic acid in the field is easily affected due to high temperature, moisture, and other environmental factors, sometimes, the efficacy in the field is reduced due to the decomposition of an active ingredient. Microcapsule technology can prolong residual efficacy *via* reducing the decomposition of an active ingredient. Methylsulfuron is a sulfonylurea herbicide, which can control a variety of grass weeds and is widely used in wheat fields globally (Zhou et al., 2019). However, if it is used improperly, such as repeated spraying and multiple spraying, or under unfavorable environmental conditions, such as rain, cooling, and flooding, severe pesticide phytotoxicity may occur after application and cause yield losses in the field, and urea or plant growth regulators (such as gibberellic acid) applied in time will reduce the impact on crops and yield (Cao et al., 2018). However, there are no reports on the microencapsulation of gibberellic acids and their detoxification effect on herbicide phytotoxicity. Microcapsules as environmentally friendly pesticide formulations play an important role. The author used the phase transfer method and *in situ* polymerization to prepare microcapsule suspensions of different types of gibberellic acid, and applied the prepared microcapsule suspensions to the field. The result showed that microencapsulation of gibberellic acid can reduce wheat herbicide phytotoxicity caused by methyl sulfuron, and this research provides a theoretical basis and data support for subsequent practical agricultural production practice in the future.

## Related Works

### Main Instruments and Reagents

#### Materials

Sodium hydroxide, 36% concentrated hydrochloric acid, urea, formaldehyde, ethyl acetate, ethyl cellulose, butyl phthalate, polyvinyl alcohol, n-dodecane, methyl oleate, xanthan gum, and ethylene glycol are all analytical grade. The above reagents were purchased from Sinopharm Chemical Reagent Co., Ltd. Tween-80 and Tween-20 are chemically pure and purchased from Hai'an Petrochemical Plant in Jiangsu Province; 30 g·L^−1^ methyldisulfuron-dispersible oil suspension agent (Shandong Bio-Biotechnology Co., Ltd.), 10% Gibberellic acid soluble powder (Zhejiang Qianjiang Biochemical Co., Ltd.), 90.3% Gibberellic acid (GA3) original medicine was provided by Sichuan Guoguang Agrochemical Co., Ltd.

#### Instruments

Instruments include JT2003B electronic balance (Yuyao Jinnuo Balance Instrument Co., Ltd.), E-100 optical microscope (Nikon Corporation), GZX-GF101 electric blast drying oven (Shanghai Yuejin Medical Instrument Co., Ltd.), WJL-602 laser particle size analyzer (Shanghai Yidian Physical and Optical Instrument Co., Ltd.), HH-2 Electric Heating Constant Temperature Water Bath (Beijing Kewei Yongxing Instrument Co., Ltd.), GC-4000 A Liquid Chromatograph (Beijing Dongxi Analytical Instrument Co., Ltd.), JP-100 Ultrasonic Cell Crusher (Shenzhen Jiemeng Cleaning Equipment Co., Ltd.), and Digital Vernier Calipers (Shijiazhuang Woma Tools Co., Ltd.).

### Preparation of 10% Gibberellic Acid Microcapsule Suspension

#### Preparation of Microcapsule Suspensions by the Phase Transfer Method

##### Aqueous Phase Preparation

Approximately 40 ml of an aqueous solution containing 0.5 g of Tween-80 and 1.5 g of PVA was prepared.

##### Organic Phase Preparation

Approximately 1 g of ethyl cellulose and 2 g of methyl oleate were weighed into a beaker, added with 5.54 g of gibberellic acid and 10 ml of ethyl acetate solution, and then stirred to completely dissolve ethyl cellulose and gibberellic acid.

##### Microcapsule Preparation

The prepared aqueous solution was poured into a three-necked flask equipped with a mechanical stirrer by adjusting the rotation speed to 1,500 r·min^−1^, controlling the water temperature at 30°C, and adding the organic phase dropwise at a speed of 3 ml·min^−1^. After dropping, stirring was continued for 20 min by slowly raising the temperature to 60°C and adjusting the rotation speed to 550 r·min^−1^, and when the ethyl acetate is evaporated, it was made up with 50 g water to obtain 10% gibberellic acid microcapsule suspension.

#### Preparation of Microcapsule Suspensions by *in situ* Polymerization

##### Preparation of the Oil Phase

About 6.18 g of gibberellic acid was weighed, dissolved in 12.0 g of ethyl acetate, and used as the oil phase.

##### Preparation of the Water Phase

In a three-neck flask equipped with a thermometer and stirring device, formaldehyde and urea were mixed according to the molar ratio of 1.75, dissolved and added with an appropriate amount of deionized water, and then added to the three-neck flask and stirred at a rate of 200 r·min^−1^. Meanwhile the pH was adjusted to 8.0 with 0.5 mol·L^−1^ sodium hydroxide solution and then warmed up to 70°C at a rate of 2°C·min^−1^, and the reaction was carried out for 1 h after warming up to obtain formaldehyde–urea prepolymer solution (Zhang, 2012). A further 8.0 g of 30% urea–formaldehyde resin prepolymer was mixed with 2.0 g of emulsifier T-20 and 21.82 g of water as the aqueous phase.

##### Preparation of Microcapsules

The oil and water phases were mixed, sheared for 60 s at 10,000 r·min^−1^, transferred to a 100-ml three-necked flask, stirred at 30°C and 800 r·min^−1^, and hydrochloric acid was used (1%). The pH was adjusted to 4.5, heated to 60°C, and stirred for 60 min. Approximately 0.1 g of xanthan gum and 2.0 g of ethylene glycol were added and stirring was continued for 30 min. Sodium hydroxide (20%) was used to adjust the pH to 9, and finally water was replenished to 50 g to obtain a 10% gibberellic acid microcapsule suspension agent with urea–formaldehyde resin as a wall material (Zhang, 2011).

### Characterization of 10% Gibberellic Acid Microcapsule Suspension

#### Determination of the Appearance and Particle Size of Microcapsule Suspension

An optical microscope was used to change the four fields of view, observe the morphology of the microcapsules, and, in the meantime, measure the average particle size of the microcapsules with a laser particle size distribution analyzer.

#### Determination of Encapsulation Efficiency

Approximately 0.05 g of the microcapsule suspension sample was accurately weighed (accurately to 0.0001 g), and an appropriate amount of methanol was added, the capsule was broken with an ultrasonic cell pulverizer, and then made up to 100 ml volume with methanol. The content of active ingredients in the microcapsule suspension was determined by liquid chromatography (Zhang, 2012).

Calculation of microencapsulation efficiency:


(1) $$\begin{array}{l} \text{Encapsulation rate }(\%)\text{=}(\text{microcapsule core material content/}\\ \text{ core material content in raw materials})\times \text{100}. \end{array}$$

Operating conditions of liquid chromatography: column temperature: room temperature; detection wavelength: 210 nm; mobile phase: methanol + water + formic acid = 40 + 60 + 0.05 (V/V); flow rate: 0.8 ml·min^−1^; injection volume: 20 μl (Ye et al., 1996).

#### Study on the Release Kinetics of Microcapsules in Organic Solvents

The prepared microcapsule suspension was filtered and dried for use. A 0.1000 g of dry sample was weighed, transferred to a 100-ml volumetric flask to break the capsule with an ultrasonic cell disruptor, and then made up to 100 ml with xylene. Liquid chromatography was used to determine the drug loading in the dried microcapsule sample.

About 0.2000 g of the sample was weighed again, transferred to a three-necked flask containing 200 ml of xylene, and stirred at 40°C and 400 r·min^−1^. Samples were taken once a day for the first 5 days, and samples were taken every 2 days from the 6th day. Aspirate 5 ml of liquid while adding 5 ml of xylene. The extracted liquid was filtered to determine the cumulative release amount at each point by liquid chromatography, and the cumulative release percentage (%) was calculated. The formula was calculated according to formula 1: (Zhang et al., 2012).

### Gibberellic Acid Microcapsule Suspension Herbicide Detoxification

#### Test Process

The wheat variety Jinmai 101 provided by the Wheat Research Institute of Shanxi Academy of Agricultural Sciences was tested. It is a semi-winter variety with a growth period of 235 days and an average plant height of about 88 cm.

The trial was conducted in Sangzi village, Hucun town, Taigu County, Jinzhong City, Shanxi province in 2018–2019. The trial field conditions are flat field, sandy loam soil, 1.57% organic, and pH 7.4. For test field preparation, the field was irrigated at 5 days before sowing to ensure good field conditions for mechanical planting. And, the wheat was sown at 24 September 2018, and the seeding amount is 300 kg·hm^−2^. The test was planted in a north-south direction *via* a randomized block design, and the plot size is 25 m^2^ (length 5 m, width 5 m, 13 rows per plot, 0.38 m pitch, and 0.50 m plot spacing), and 1.00 m width walkway between replications, and even crop vigor among four replications. After returning to the green (21 March 2019), methyldisulfuron was applied at 30 g·L^−1^ and the dosage was 30 g ai·ha^−1^. Following the finding of methyldisulfuron phytotoxicity symptom, such as yellowing of leaves or the weakness of plants or reduced plant height or stunted growth (26 March 2019) on wheat, gibberellic acid microcapsule suspension was applied to wheat with four replications. The treatment list is presented in Table 1.

**Table 1:** Concentration of the reagents used in the test field.

**No**.	**Pharmacy**	**Active ingredient dosage (ml·ha^**−1**^)**	**Remarks**
1	10% gibberellic acid microcapsule suspension (Microcapsule 1)	250	Prepared by the phase transfer method
2		500	
3		750	
4	10% gibberellic acid microcapsule suspension (Microcapsule 2)	250	Prepared by *in situ* polymerization
5		500	
6		750	
7	10% gibberellic acid soluble powder	250	—
8		500	
9		750	
10	Untreated check	–	-

*Microcapsule 1 represents the formulation prepared by the phase transfer method and microcapsule 2 represents the formulation prepared by the in situ polymerization method, and so on*.

#### Survey of the Results

Samples were collected before and at 21 days after the application of the microcapsule suspension, five points per plot, and five plants per point. The height of each plant was investigated, and the plant height growth and growth promotion rate were calculated. The calculation formula was as follows: formulas (2) and (3). Meanwhile, samples were collected before harvest once again, five points per plot, and five plant per point. Ear length, ear diameter, single ear weight, and 1,000-kernel weight were investigated, and the yield of each plot was assessed.


(2) $$\begin{array}{l} \text{Plant height growth =plant height }(\text{cm})\text{ after}\\ \text{ medicine-plant height }(\text{cm})\text{ before medicine}. \end{array}$$

#### Data Analysis

Variance analysis on the above mentioned index traits was analyzed by the SPSS data processing system, and multiple comparisons were performed to analyze the differences between the different agents *via* a new complex range method.

## Results and Discussion

### Determination of Performance Indicators of Microcapsule Suspensions

#### Determination of the Appearance and Particle Size of Microcapsule Suspensions

**Figure 1:**
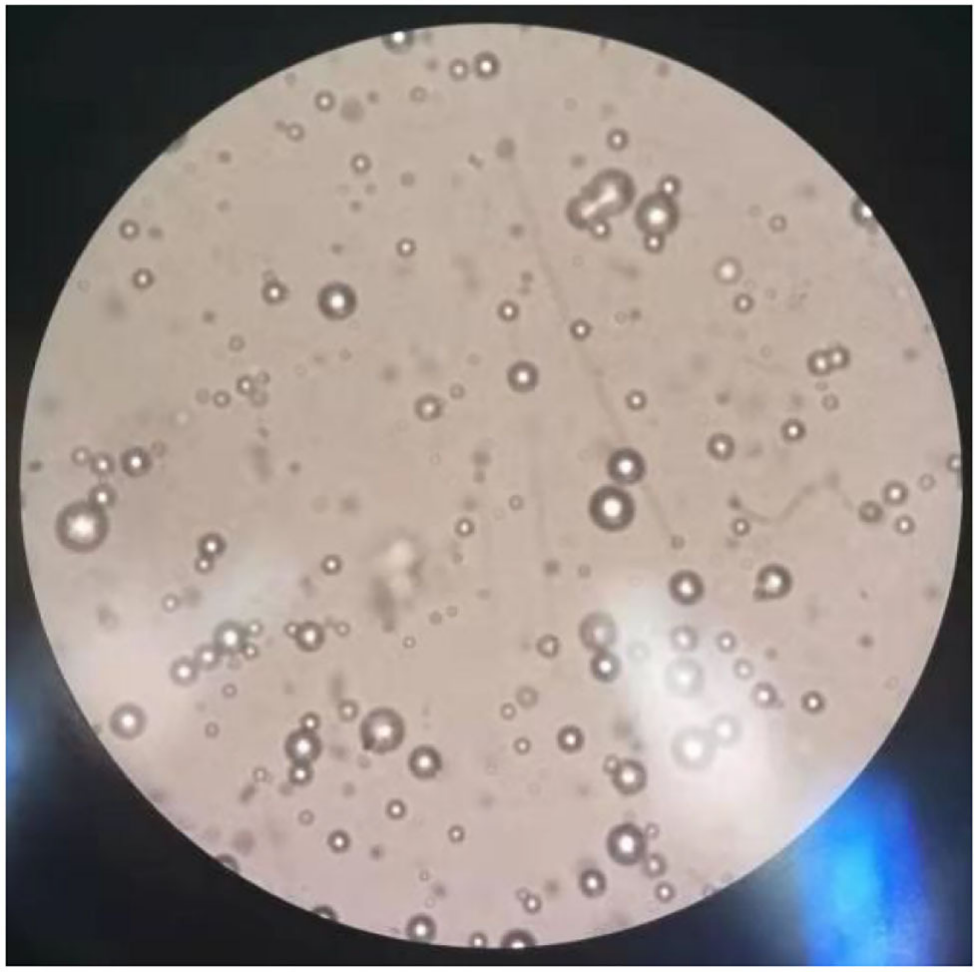
Microscopic images on microcapsule particle size prepared by the phase transfer method (100×).

**Figure 2:**
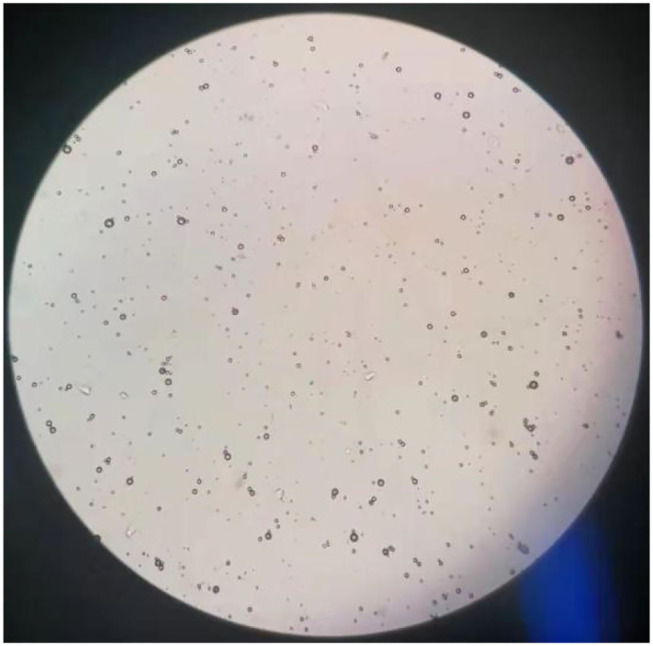
Microscopic images on microcapsule particle size prepared by *in situ* polymerization (100×).

**Figure 3:**
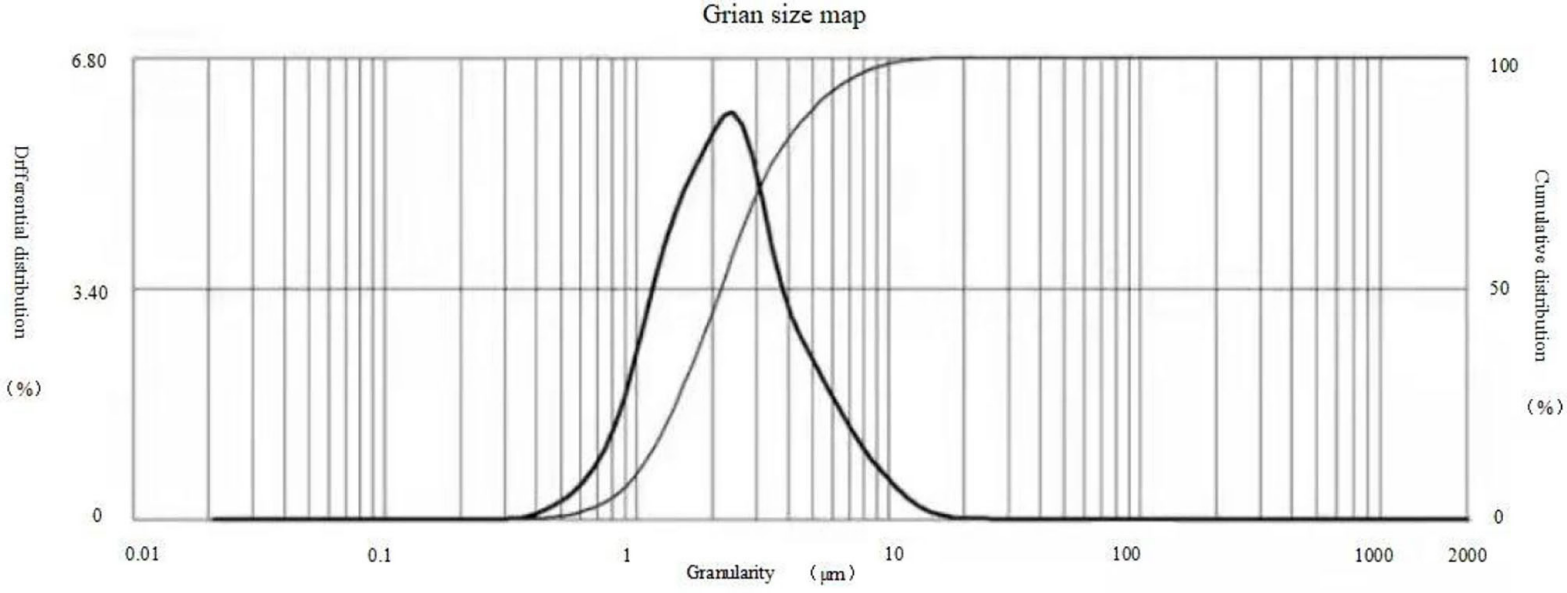
Particle size distribution of microcapsule 1.

**Figure 4:**
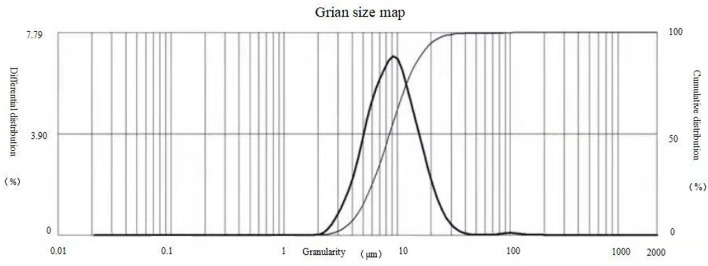
Particle size distribution of microcapsule 2.

The results of observations of microcapsules 1 and 2 using an optical microscope are shown in Figures 1, 2. The particle size measured by the laser particle size distribution analyzer showed that: for microcapsule 1, D_3_ is 0.697 μm, D_10_ is 0.990 μm, D_50_ is 2.136 μm, D_90_ is 5.201 μm, and D_97_ is 7.987 μm, δ = 1.971; for microcapsule 2, D_3_ is 3.241 μm, D_10_ is 4.365 μm, D_50_ is 8.547 μm, D_90_ is 16.782 μm, and D_97_ is 22.946 μm, δ = 1.453; particle size. Distribution diagrams are shown in Figures 3, 4. Comprehensively, the result showed that microcapsule 1 had a smaller particle size, worse monodispersity, and wider particle size distribution compared with microcapsule 2. This phenomenon occurs during the microcapsule preparation process. The selection of the emulsifier is related to the selection of a suitable emulsifier when preparing the aqueous phase, which can emulsify the oil phase into fine oil droplets and disperse uniformly in the aqueous phase.

#### Determination of Encapsulation Efficiency

The encapsulation rate of microcapsules 1 and 2 was 86.9% and 91.2%. The results showed that the encapsulation rate of microcapsules prepared by the *in situ* polymerization method is high. It is possible that microcapsule 2 is formed by encapsulating the active ingredients after polymerization of the capsule wall monomer, the formed capsule is denser; while microcapsule 1 is a capsule prepared by the physical method, the phase transfer occurs in the capsule wall. Due to the influence of the volatilization rate of organic solvents, the conditions are difficult to grasp, and the cysts formed are not uniform.

#### Study on the Release Kinetics of Microcapsules Into Organic Solvents

**Figure 5:**
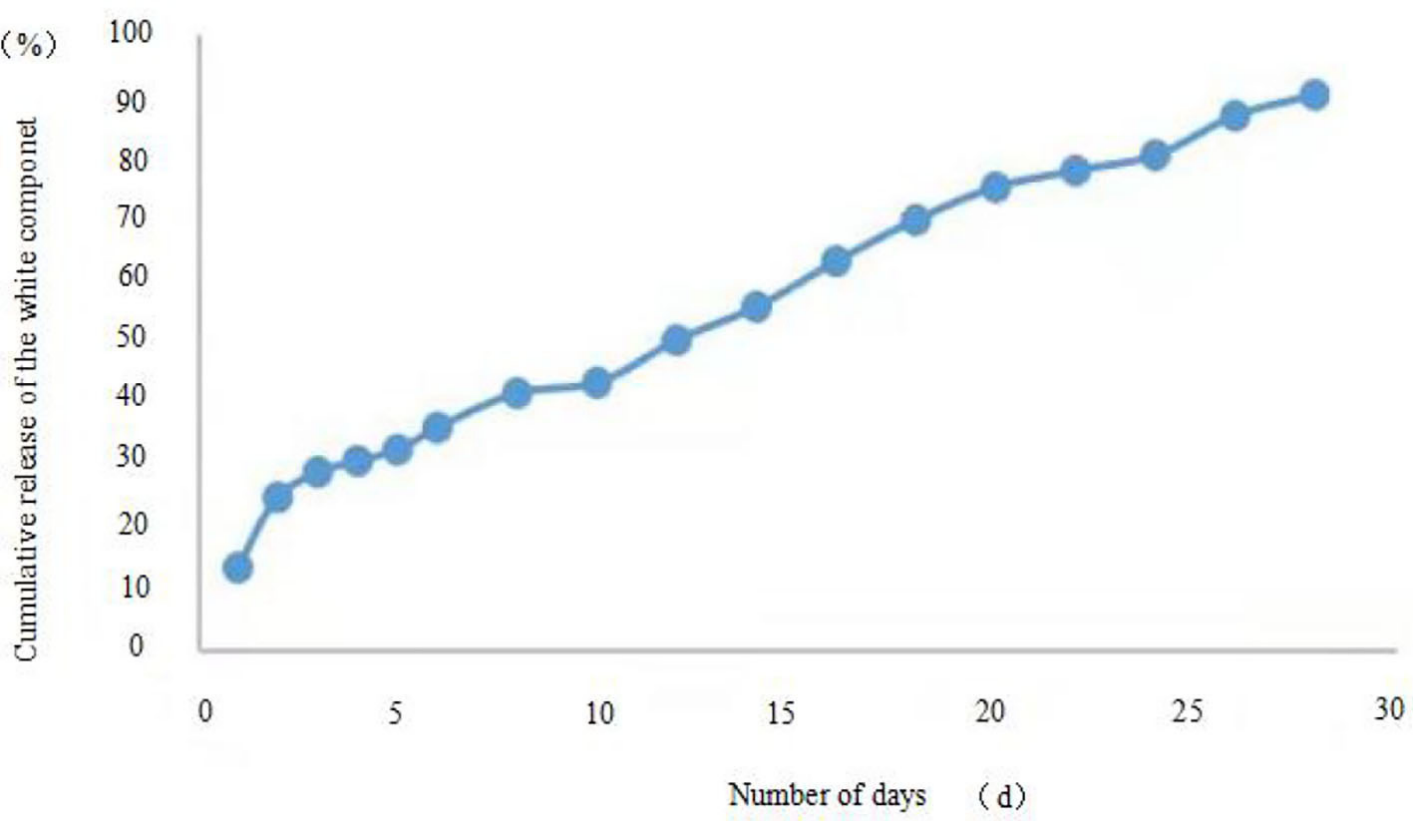
Microcapsule 1 release curve.

**Figure 6:**
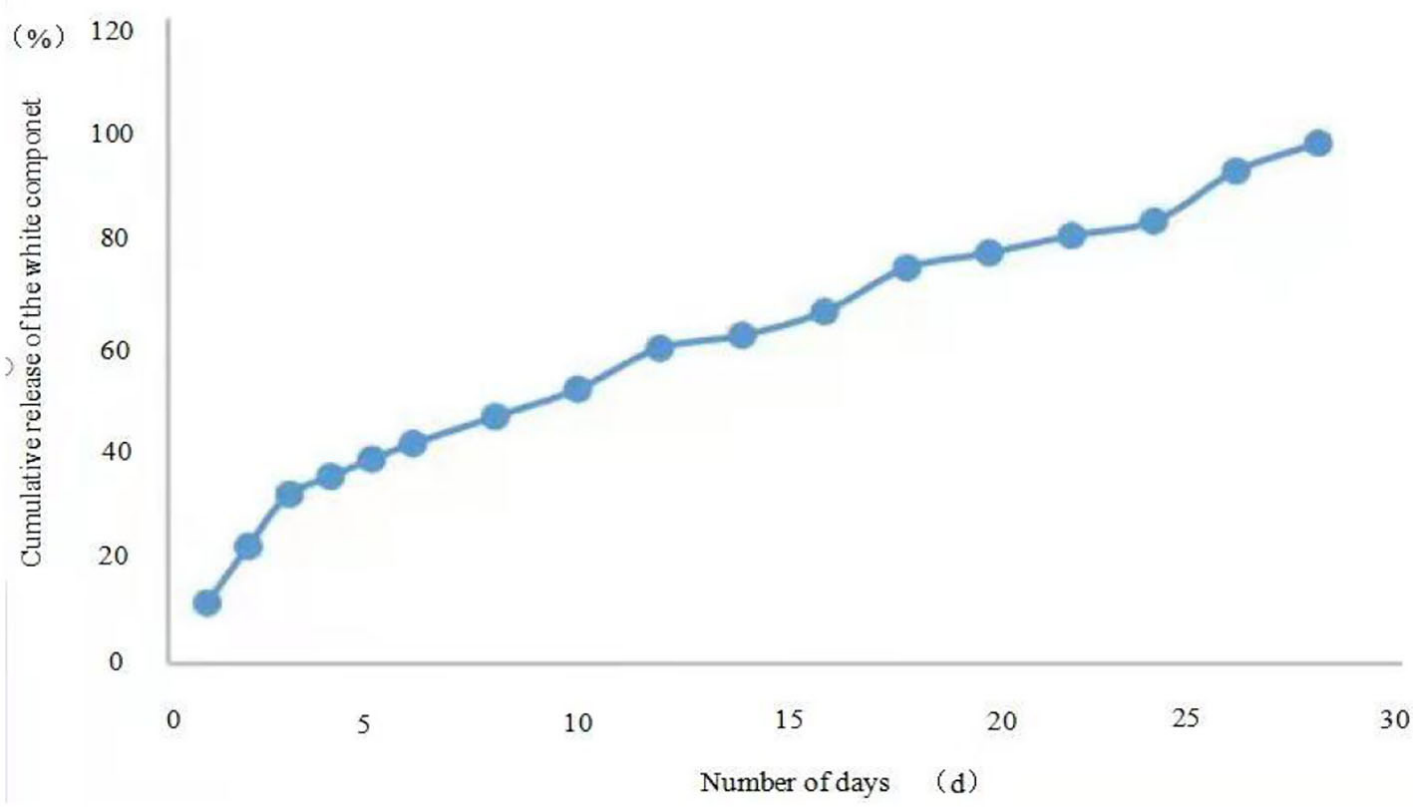
Microcapsule 2 release curve.

The release profiles of the microcapsules prepared by the two methods are shown in Figures 5, 6. For microcapsule 1, the release rate on the 1st day was 13.4%, and the cumulative release rate in the first 5 days was 32.5%, indicating that the microcapsules had a rapid release process in the early stage. From 0 to 5 days, by fitting the kinetic equation with time and cumulative release, the zero-order release kinetic equation of microcapsule 1 is *y* = 5.6× + 10.45 (*R*^2^ = 0.8659, *Q* is the cumulative release percentage), and the first-order release kinetic theoretical equation is Ln(1 – *Q*) = −0.0725× + 4.5022 (*R*^2^ = 0.8828). The *R*^2^ value of the zero-order release kinetic equation is lower than that of the first-order release kinetic equation. This means that the release of microcapsule 1 complies with the first-order kinetic equation from 0 to 4 days, and the release behavior of the active ingredients in the microcapsules has entered a stable phase from the 5th day. From 5 to 28 days, the zero-order release kinetic equation is *y* = 2.5519× + 20.792 (*R*^2^ = 0.989), and the first-order release kinetic equation is Ln(1 – *Q*) = −0.0792× + 4.7626 (*R*^2^ = 0.9581). The *R*^2^ value of the zero-order release kinetic equation is greater than that of the first-order release kinetic equation, which means that the release of microcapsule 1 fits the zero-order kinetic equation from 5 to 28 days.

Similarly, the first-order release kinetic equation of microcapsule 2 released in 0–4 days is Ln (1–*Q*) = −0.1054× + 4.5772 (*R*^2^ = 0.9676), and the zero-order kinetic equation is *y* = 8.02× + 4.7 (*R*^2^ = 0.9568), the release from 5 to 28 days the first-order kinetic equation is Ln(1–*Q*) = −0.0998× + 4.8952 (*R*^2^ = 0.8367), the zero-order kinetic equation is *y* = 2.4675× + 26.422 (*R*^2^ = 0.993), which means that the release of microcapsule 2 from 0 to 4 days conforms to the first-order kinetic equation, and the release of microcapsules in 5–28 days conforms to the zero-order kinetic equation.

### Research on Detoxification

#### Effect of Pesticides on Wheat Growth

The effects of the different pesticides on wheat growth are shown in Table 2. At 14 days after application, plant height in all gibberellic acid treatments is significantly higher than that in untreated checks, and a clear dosage response was found in different gibberellic acid treatments. Meanwhile, there are no significant differences between microcapsules 1 and 2 at the same dosage, which are significantly lower compared with gibberellic acid soluble powder treatment at the same dosage. At 28 days after application, plant height in all gibberellic acid treatments is still significantly higher than that in untreated checks. Meanwhile, plant height in microcapsule treatments is significantly higher than that in gibberellic acid soluble powder treatment at the same dosage due to the longer residual control from the microcapsule compared with the soluble powder formulation, in terms of comparison of two types of microcapsule, plant height in microcapsule 2 @ 750 ml/ha is significantly higher than microcapsule 1 @ 750 ml/ha, and plant height in microcapsules 1 and 2 @ 250–500 ml/ha is comparable to each other at the same dosage.

**Table 2:** Detoxification effects of different pesticides on wheat.

**Treatments**	**Amount of active ingredients (ml·ha^**−1**^)**	**Growth height (cm)**		**Growth promotion rate**	
		**14 days after application**	**28 days after application**	**14 days after application**	**28 days after application**
Microcapsule 1	250	10.28d	31.96e	11.14d	19.50e
	500	13.48c	33.93cd	45.77c	26.88cd
	750	15.00b	35.37b	62.18b	32.26b
Microcapsule 2	250	10.70d	31.18e	15.71d	16.58e
	500	13.48c	34.71bc	45.77c	29.80bc
	750	15.24b	37.78a	64.79b	41.26a
10% gibberellic acid soluble powder	250	12.96c	28.42f	40.13c	6.26f
	500	14.80b	31.07e	59.99b	16.17e
	750	18.65a	33.42d	101.70a	24.97d
Untreated Check	–	9.25e	26.74 g		

*The meaning of the letters a–d represents the significance of the difference, and the different letters indicate that the results are significantly different from each other*.

#### Effect of Pesticides on Wheat Yield

The effects of spraying different pesticides on wheat yield are shown in Table 3. A clear dosage response was found in all the assessment factors for all the three gibberellic acid formulations. Yield performance from three gibberellic acid formulations @ 250 ml/kg is comparable with the untreated check due to comparable or slightly better performance in the assessed factors, such as ear diameter, ear length, single ear weight, and 1,000-grain weight, from different gibberellic acid formulations compared with the untreated check, and a significantly higher yield from three gibberellic acid formulations @ 500–750 ml/kg with the untreated check due to significantly better performance in the assessed factors, such as ear diameter, ear length, single ear weight, and 1,000-grain weight. Meanwhile, the yield of both microcapsules @ 750 ml/ha is significantly higher than gibberellic acid soluble powder @ 750 ml/ha, which is driven by 1,000-grain weight and single ear weight, and all the assessed factors of microcapsules 2 @ 750 ml/ha are significantly better than gibberellic acid soluble powder @ 750 ml/ha, and the assessed factors of microcapsule 1 @ 750 ml/ha are better than or comparable with gibberellic acid soluble powder @ 750 ml/ha. Comprehensively, microcapsule 2 provided the best effect in the field especially on yield, followed by microcapsule 1, which is better than the control agent. This shows that the microcapsules can significantly increase the yield of wheat through yield loss reduction due to the damage caused by pesticides (Wang et al., 2000).

**Table 3:** An effect of the different pesticides on wheat yield.

**Elixir**	**Amount of active ingredient (ml ·ha^**−1**^)**	**Ear diameter (cm)**	**Ear length (cm)**	**Single spike weight (g)**	**1,000 kernel weight (g)**	**Yield (kg·hm^**−2**^)**	**Yield increase (%)**
Microcapsule 1	250	1.18ef	7.51d	2.22cde	40.01e	6893.45ef	3.45ef
	500	1.23cd	7.72bc	2.39b	43.23cd	7463.73bcd	12.01bcd
	750	1.30ab	7.89ab	2.59a	45.71ab	8624.31a	29.43a
Microcapsule 2	250	1.21de	7.53d	2.24cd	41.71de	7073.54def	6.16def
	500	1.25bc	7.78ab	2.38b	44.10bc	7763.88b	16.52b
	750	1.32a	7.94a	2.67a	46.75a	8824.41a	32.43a
10% gibberellic acid soluble powder	250	1.15f	7.34e	2.19de	37.52f	6683.34f	0.30f
	500	1.21de	7.60cd	2.31bc	39.83e	7243.62cde	8.71cde
	750	1.26bc	7.75bc	2.40b	41.57de	7623.81bc	14.41bc
Blank control	–	1.08 g	7.18f	2.12e	35.25 g	6663.33f	–

*Different lowercase letters after the same column of data indicate a significant difference of 5%*.

## Discussion

### Characterization of Pesticide Microcapsule Release Properties

Microcapsule suspensions, as a new type of controlled released formulation, have the outstanding advantages of prolonging the residual efficacy from the active ingredient through slow release (Liu and Xie, 2005). Release is an important indicator, which can directly reflect the effect of sustained release and durability of microcapsules, and it is of great significance for evaluating and using pesticide microcapsules (Zhao et al., 2007). Meanwhile, the release of the microcapsules has a significant impact by means of environmental conditions, such as temperature, pH, and moisture. And, the release mechanism is usually done through physical routes, such as dissolution, diffusion, penetration, and ion exchange, and chemical pathways, such as active ingredients or enzyme degradation (Chen, 2015; Xue, 2019), normally, there are two phases in the release process, which are rapid release and slow release phase. In the rapid release phase, an active component on the outer wall of the capsule may not be coated, resulting in a large amount of initial release, which is consistent with the “burst release effect” of the initial release of microcapsules (Jegat and Taverdet, 2000). In the slow-release phase, the active ingredient diffuses through the wall of the capsule over time, the release is gentle and the cumulative release amount increases slowly; through these two phases, the microcapsules can be guaranteed to have a rapid and sustained effect. During the experiment, the microcapsules were placed in an organic solvent to simulate the actual release of the microcapsules through diffusion. There are two stages in the release process of microcapsules 1 and 2, which meet the characteristics of slow release of microcapsules. The mechanism and characterization of the release properties of pesticide microcapsules prepared from different capsule wall materials need to be further explored.

### Comparison of Field Effects of Different Methods for Preparing Microcapsules

This experiment verifies that the microcapsules prepared by the two different methods have different effects in their actual use. The specific performance is that the microcapsules prepared by the *in situ* polymerization method are more effective than the microcapsules prepared by the phase transfer method, both of which are higher than soluble powder. The better performance should be caused by a smaller particle size of microcapsule 2 compared with microcapsule 1. The smaller particle size means that the microcapsule is easier to attach to the surface of the crop and not easy to fall. Meanwhile, the emulsifier and processing technology are also related; on the other hand, the release is faster, and the amount released after 28 days is higher in microcapsule 2 than in microcapsule 1. Through the release of microcapsules, gibberellic acid can be continuously released into the environment, to extend the efficacy in promoting crop growth, and continuously increase crop tolerance against herbicide damage. However, the efficacy at 14 days is not as good as the soluble powder formulation, which may be caused by the microcapsules that have not yet been completely released. The control agent has a higher amount of gibberellic acid active ingredients on the surface of wheat than the microcapsules, at 28 days, the efficacy is significantly better than the soluble powder formulation, which demonstrates the longer durability of the microcapsules and the advantages of resisting the effects of the natural environment on the active ingredients.

## Conclusion

Two gibberellic acid microcapsule suspensions prepared by the phase transfer method and the *in situ* polymerization method have a uniform particle size distribution. The encapsulation efficiency and release characteristics meet the requirements of the microcapsules. The preparation method and characterization of microcapsules can provide a theoretical basis and supporting data for their production and practical applications, and have a certain application value. At the same time, the use of 10% gibberellic acid microcapsules and 10% gibberellic acid soluble powder can reduce herbicide damage to crop growth caused by methyldisulfuron in wheat. The two types of microcapsules have a different effect. Through the indexes of microcapsule particle size, encapsulation rate, release rate, and the effect on wheat growth, it was found that the performance of the microcapsules prepared by the *in situ* polymerization method was better than that of the microcapsules prepared by the phase transfer method, and the microcapsules can prolong the product residual efficacy in the field *via* controlled release; therefore, the *in situ* polymerization method was recommended for practical production.

## Data Availability Statement

The raw data supporting the conclusions of this article will be made available by the authors, without undue reservation.

## Author Contributions

BZ was responsible for the framework design and topic selection of this whole manuscript. GZ designed and implemented solutions and wrote the article. JW and LL were responsible for experimental verification and data sorting. TM was responsible for data checking. YC is in charge of proofreading articles. All authors approved this manuscript for publication.

## Funding

This work was funded by the Shanxi Department of Science and Technology, Shanxi Science and Technology Foundation Platform Project (201605D121024), and Shanxi Agricultural University Science and Technology Innovation Project (412579).

## Conflict of Interest

YC was employed by Bayer Crop Science (China) Co., Ltd. The remaining authors declare that the research was conducted in the absence of any commercial or financial relationships that could be construed as a potential conflict of interest.

## Publisher's Note

All claims expressed in this article are solely those of the authors and do not necessarily represent those of their affiliated organizations, or those of the publisher, the editors and the reviewers. Any product that may be evaluated in this article, or claim that may be made by its manufacturer, is not guaranteed or endorsed by the publisher.
